# Characterization of 3-Hydroxyeticyclidine (3-HO-PCE) Metabolism in Human Liver Microsomes and Biological Samples Using High-Resolution Mass Spectrometry

**DOI:** 10.3390/metabo13030432

**Published:** 2023-03-16

**Authors:** Islam Amine Larabi, Delphine Joseph, Camille Lesueur, Jean-Claude Alvarez

**Affiliations:** 1 Department of Pharmacology and Toxicology, Raymond Poincaré Hospital, AP-HP, 92380 Garches, France; camille.lesueur@hotmail.fr (C.L.);; 2 UVSQ, Université Paris-Saclay, Inserm U1018, CESP, Équipe MOODS, MasSpecLab, 78180 Montigny-le-Bretonneux, France; 3 Université Paris-Saclay, CNRS, BioCIS, 91400 Orsay, France

**Keywords:** 3-Hydroxyeticyclidine, 3-HO-PCE, ketamine, arylcyclohexylamine, new psychoactive substances, hair, high-resolution mass spectrometry, HLM

## Abstract

3-Hydroxyeticyclidine (3-HO-PCE) is a ketamine derivative that produces dissociative, hallucinogenic, and euphoric effects when consumed, but little is known about its pharmacological properties, metabolism, and toxicity compared to other designer ketamine analogs. To address this gap in knowledge, this study explored for the first time the metabolism of 3-HO-PCE. Based on this investigation, it is hypothesized that combining the use of Human Liver Microsomes (HLM) as an In vitro model with urine and hair samples from drug users may enable the identification of key analytes that can extend the detection window of 3-HO-PCE, particularly in cases of overdose. The analysis identified 15 putative metabolites, 12 of which are produced through phase I metabolism involving *N*-dealkylation, deamination, and oxidation, and 3 through phase II *O*-glucuronidation. The metabolism of 3-HO-PCE is similar to that of O-PCE, another designer ketamine of the eticyclidine family. The study identified M2a and hydroxy-PCA as reliable biomarkers for untargeted screening of the eticyclidine family in urine and hair, respectively. For targeted screening of 3-HO-PCE, M10 is recommended as the target analyte in urine, and M5 shows promise for long-term monitoring of 3-HO-PCE using hair analysis.

## 1. Introduction

The introduction of novel psychoactive substances (NPS) continues to escalate, and these potent and dangerous substances are a public health concern. The latest European Drug Report highlights the detection of over 830 NPS in the European drug market, with synthetic cannabinoids and cathinones constituting nearly 60% of the seizures and aryl-cyclohexylamines (ketamine analogues) accounting for a further 10% [1]. Furthermore, the use of hallucinogens, which accounted for 15% of the 1047 NPS reported to the United Nations Office on Drugs and Crime (UNODC) in 2020, has become a matter of increasing concern [2].

Ketamine has traditionally been utilized as an anesthetic, analgesic, sedative, and in the treatment of chronic pain syndromes. However, its distinctive pharmacodynamic characteristics as have sparked interest in its potential use for depression treatment [3].

Recreational use of ketamine as a dissociative drug has gained popularity in certain regions such as Asia (India and China) and Europe (Belgium, France, and The Netherlands). According to the UNODC, 37 of the 86 countries reporting ketamine seizures between 2008 and 2020 were in Europe, while 25 were in Asia [2,4]. 

Dissociative anesthetics, including ketamine, phencyclidine, or 2F-DCK belong to a diverse group of hallucinogens that also encompasses traditional hallucinogens such as LSD, dimethyltryptamine, and 5-MeO-DMT. The mechanism of action of traditional hallucinogens involves the activation of serotonin receptors, which alter the perception of reality and evoke synesthesia. In contrast, dissociative anesthetics exhibit both stimulating and dissociative properties by inhibiting the reuptake of dopamine, norepinephrine, and serotonin, as well as blocking NMDA (N-methyl-D-aspartate) receptors. This results in a sensation of detachment and dissociation from both the self and the surroundings [2,3]. N-Methyl-D-aspartate (NMDA) receptor antagonism is implicated in mediating, at least in part, the subjective and mind-altering effects of many compounds classified as dissociatives [5].

The subjective effects of dissociative drugs are complex and highly dose dependent. At low doses, typically induce a state of intoxication subjectively comparable to ethanol. Other common effects include euphoria, tactile, visual and auditory hallucinations, altered thought patterns, paresthesia, depersonalization, and derealization. Higher doses often lead to full dissociation from the sensory environment, anesthesia, catalepsy, and motor impairment [5]. This may lead to acute intoxications and even fatalities as reported in several studies [6,7,8].

A wide range of compounds with similar or enhanced effects have been covertly produced by modifying the nature of the functionalizing groups of ketamine. 

It is worth noting that the impact of the β-keto group on the structure-activity relationships of these compounds compared to their arylcyclohexylamine counterparts has not been thoroughly investigated. Nevertheless, research suggests that the dissociative effect is still present in the absence of the β-keto group. Additionally, substitutions of the chlorine atom in ketamine with other halogens like fluorine in 2F-DCK or hydrogen in deschloroketamine (DCK) seem to maintain the dissociative effects. It is important to consider, however, that the intensity of the dissociative effect may differ depending on the specific compounds being studied [5,7,9,10].

**Figure 1:**
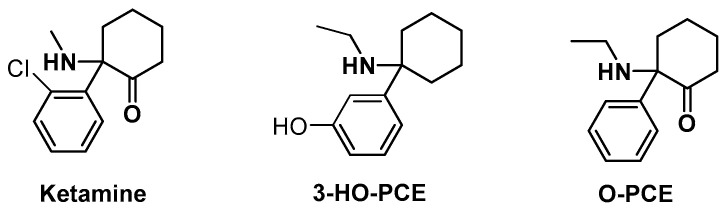
Structures of ketamine, 3-HO-PCE, and O-PCE.

Ketamine derivatives can be divided into three main subfamilies: ketamine-like, phencyclidine-like (PCP-like) and eticyclidine-like (PCE-like) molecules [11]. Among the PCE-like family, 3-hydroxyeticyclidine, commonly referred to as “3-hydroxy PCE” or “3-HO-PCE,” emerged on the market of research chemicals in the 2010s [4]. 3-HO-PCE is a derivative of aryl amino cyclohexane, sharing the core structure of 1-phenyl-1-amino cyclohexane with ketamine. Ketamine features a chlorine atom in the ortho position of the phenyl ring, a methyl group on the amine function and a vicinal carbonyl function on the cyclohexane moiety. 3-HO-PCE differs in that it has an ethyl group instead of the methyl group on the amine function, and the phenyl ring substituted by a hydroxy group in meta position instead of chlorine atom in ortho position, and the absence of the ketone function on the carbocycle. In turn, O-PCE exhibits the dehydroxylated structure of 3-HO-PCE by replacing the phenol nucleus by an unfunctionalized phenyl ring, and a ketone function on the cyclohexane group [12] (Figure 1).

There is a lack of data on the pharmacological properties (pharmacokinetic and pharmacodynamic), metabolism and toxicity of 3-HO-PCE, but early reports speculated that its dissociative effects profile has been characterized as “lying halfway between 3-MeO-PCP and 3-MeO-PCE.” [13].

Comprehending metabolic pathways helps to pinpoint relevant biomarkers of drug usage. Detecting metabolites in clinical and forensic settings is a more dependable way of confirming drug intake since the presence of the parent drug alone could be due to external contamination [14,15]. Additionally, metabolites with high abundance and/or a long elimination half-life extend the detection window in biological samples [16]. Moreover, identification of metabolites is interesting since some of them could also have a pharmacodynamic effect. Lastly, studying drug metabolism can provide a toxicological risk assessment by investigating drug-drug interactions that may cause some reported adverse effects. Even though human research is not feasible due to ethical considerations, various in vivo and in vitro models are used as a substitute [17]. The use of pooled human liver microsomes (pHLM) is a prevalent and well-established model for studying drug metabolism, as it allows for the determination of intrinsic hepatic clearance. This model permits the examination of both phase I and certain phase II metabolites (e.g., glucuronides), and the identification of their chemical structure and kinetics, primarily through ultra-performance liquid chromatography (UPLC) coupled to high-resolution mass spectrometry (HRMS) [18,19,20,21,22,23,24,25]. 

Currently, there is a lack of data on the metabolic pathways of 3-HO-PCE to allow the identification of specific biomarkers that could be used as reference in forensic screening. This study aims to determine the metabolic profile of 3-HO-PCE and to identify new biomarkers for its detection in human samples. It is based on the exploration of the 3-HO-PCE HLMs metabolism and its comparison to urine and hair samples from 3-OH-PCE consumer using ultra-performance liquid chromatography (UPLC) coupled to high-resolution mass spectrometry (HRMS).

## 2. Materials and Methods

The structure and the quality of 3-HO-PCE powder, which was procured online and obtained from a drug user during a harm reduction session, was assessed through Nuclear Magnetic Resonance (NMR), as per a previously published protocol [26]. The sample exhibited a purity higher than 95% with no adulterants detected and was then employed as a reference standard for the in vitro investigation.

Sigma-Aldrich (France) provided alamethicin (Trichoderma viride), tris hydrochloride (HCl), anhydrous magnesium chloride, uridine 5′-diphosphoglucuronic acid tris (UDPGA), glucose-6-phosphate dehydrogenase (G6PDH), D-glucose 6-phosphate (G6P), and the reduced form of nicotinic acid adenine dinucleotide phosphate (NADPH, 98%). Biopredic (La Bretèche, Saint Grégoire, France) supplied a solution of pooled human liver microsomes (pHLM) derived from 17 donors (7 females and 10 males) aged 31–78 years, which was titrated at 25 mg of protein/mL and stored at −80 °C until use. 

Chemicals such as acetonitrile (ACN), chloroform, isopropanol, ethyl acetate, formic acid, hexane, and methanol (MeOH) of mass spectrometry (MS) or high-performance liquid chromatography (HPLC) grades were purchased from Sigma Aldrich (Paris, France); while sodium carbonate and sodium bicarbonate were supplied by Prolabo (Paris, France). Ultrapure water (18 MΩ) was obtained through ultrafiltration using a Q-Pod (Millipore Corp., Molsheim, France). Formate buffer, a 2 mM solution of ammonium formate in 0.1% formic acid (solvent A), was prepared and stored at +4 °C away from light for a maximum of one week.

### 2.1. In Vitro Study Using Human Liver Microsomes (HLM)

#### 2.1.1. Sample Pretreatment

According to our previously established procedure [16], 100 µL of thawed pHLM (20 mg/L) were activated on crushed ice using 250 µL of a 50 µM alamethicin solution mixed with 150 µL of Tris-HCl-MgCl2 buffer solution (pH 7.4). Then, 50 µL of this mixture were combined with 50 µL of a 100 µM 3-HO-PCE solution in MeOH, which had been previously evaporated to dryness under gentle nitrogen stream. 50 µL of cofactor solution (5 mM UDPGA, 3.2 mM NADPH, 8.5 mM G6P, and 0.5 U/mL G6PD) prepared in the same buffer (Tris-HCl-MgCl_2_) was added. The enzymatic reaction was carried out at 37 °C for 2 h and terminated by adding 200 µL of MeOH. Samples were centrifuged at 4 °C for 10 min at 14,000 rpm, and 10 µL of the supernatant were injected into the LC-HRMS system. Two additional blank samples were concurrently analyzed: (1) blank pHLM-1, which was a reagent blank (pHLM + cofactors) without the substrate (3-HO-PCE), used to subtract the matrix background signal, and (2) blank pHLM-2, which contained 3-HO-PCE and pHLM without cofactors to prevent misinterpretation of non-enzymatically formed compounds during the drying and/or incubation process.

#### 2.1.2. Instrument Conditions

A LC-HRMS method described in a previous publication [27] was used in the present study. A phenyl-hexyl (PH) column (100 mm × 2.1 mm i.d., 2.6 μm particle size, Thermo Fisher Scientific, Waltham, USA) was kept at 40 °C, and elution was performed in gradient mode using a mixture of water/ammonium formate (2 mM)/0.1% formic acid (solvent A) and ACN/MeOH (50:50, *v*/*v*)/1% water/0.1% formic acid (solvent B) at a flow rate of 500 µL/min. The total run time was 20 min, with a gradient of solvent B from 1–99% in 17.5 min.

The Q-Exactive orbitrap accurate mass spectrometer (Thermo Fisher Scientific, Waltham, MA, USA) with a heated electrospray ionization (HESI) probe was used to detect compounds in positive-ion mode. The data were acquired in Data Dependent Acquisition (DDA) mode, consisting of one full MS scan and up to five data-dependent MS/MS scans (full MS/ddMS2). The five ions with the most intense signal in the full MS scan (intensity threshold, 1.0 × 105) triggered a specific MS/MS spectrum. An exclusion list of mobile phase raw data was created to achieve the lowest limit of identification (LOI).

The full MS settings were as follows: resolution, 70,000 FWHM; scan range, *m*/*z* 50–650; automatic gain control (AGC) target, 1.0 × 10^6^; and maximum injection time (MIT), 50 ms. The ddMS2 settings were: resolution, 17,500 FWHM; AGC target, 1.0 × 10^5^; MIT, 200 ms; isolation mass window, 1 *m*/*z*; normalized collision energy (NCE), 55%; dynamic exclusion time, 9 s. 

The mass spectrometer was calibrated daily over a mass range of 50–2000 *m*/*z* using Xcalibur software (v.4.0, Thermo Fisher Scientific, Waltham, MA, USA).

### 2.2. Analysis of Biological and Non-Biological Samples from a Drug User

#### 2.2.1. Sample Collection 

As part of a harm-reduction session, a 22 years old polydrug user provided, after signing a consent form, urine, hair, and 3-HO-PCE powder samples to a practitioner for routine toxicology testing, which were subsequently sent to our laboratory. However, the exact amount of 3-HO-PCE consumed and the time of the last use were not disclosed.

#### 2.2.2. Urine Pretreatment 

To prepare the urine samples for analysis, 200 µL of both non-hydrolyzed and hydrolyzed urine (using β-glucuronidase per the supplier’s protocol) were subjected to liquid-liquid extraction using 4 mL of hexane/ethyl acetate (1/1, *v*/*v*). After 10 min of agitation and 10 min of centrifugation at 14,000 rpm, the organic layer was separated, and the aqueous phase was subjected to a second extraction using 4 mL of chloroform/isopropanol (4/1, *v*/*v*). The combined organic layers were then dried, reconstituted in 80 µL of solvent A, and finally 10 µL of the solution was injected into the system for analysis.

#### 2.2.3. Hair Pretreatment 

A hair sample measuring 4 cm in length was taken from the patient, which corresponds to a period of 4 months before the time of sampling, assuming an average hair growth rate of 1 cm per month [28]. The entire segment was analyzed as a whole, without any further segmentation.

The segment was decontaminated using two dichloromethane baths, ground into a fine and homogeneous powder using a ball mill (MM200, FicherScientific, Illkrich, France), and 20 mg were incubated in 1 mL of phosphate buffer at pH 5.0 at 95 °C for 10 min before undergoing the same extraction procedure as that used for the urine sample. Briefly, liquid–liquid extraction was performed with 4 mL of hexane/ethyl acetate (*v*/*v*: 1/1). After agitation and centrifugation, the organic phase was recovered and evaporated to dryness. The residue was reconstituted in 80 μL of solvent A and 10 μL were injected in the analytic system [4].

#### 2.2.4. Recovered Material

An aliquot of 10–30 mg of 3-HO-PCE powder was dissolved in deuterated methanol for NMR analysis.

#### 2.2.5. Instrument Conditions

- Urine

Urine was analyzed by LC-HRAM according to the above-described procedure applied to pHLM.

- Hair

Hair was analyzed by LC-HRAM according to the procedure applied to pHLM.

- Recovered 3-HO-PCE powder

Various NMR experiments were conducted, including 1H, 13C, and DEPT 13C NMR experiments. Additionally, advanced two-dimensional (2D) NMR techniques were employed, such as correlated spectroscopy (COSY), heteronuclear single quantum correlation (HSQC), and heteronuclear multiple-bond correlation (HMBC). All NMR spectra were acquired using Brüker Avance spectrometers, which operate at a frequency of 300 MHz and are equipped with a BBFO probe (Supplementary Material Figure S1).

### 2.3. Post-Processing of HRAM Data 

The raw data obtained from the study were processed using Compound Discoverer 3.2 software (Thermo Fisher Scientific, Waltham, MA, USA), which utilized a metabolic workflow to identify potential metabolites. This workflow involved phase I biotransformation reactions, such as oxidation, reduction, and hydrolysis, as well as phase II reactions, such as glucuronide conjugation. The 3-HO-PCE chemical structure was uploaded in a .mol file format, and data was processed after background subtraction. Metabolites were predicted based on their exact mass, elemental composition, isotopic pattern, and ddMS2 spectra. The *m*/*z* shifts from the parent compounds fragmentation were used to localize metabolically transformed moieties. Only features triggering MS2 experiments were considered, and the mass error between theoretical and experimental exact mass had to be within ±5 ppm. The resulting annotated spectra were manually reviewed to generate a final metabolic profile using ChemDraw 20.1 software (PerkinElmer, Waltham, MA, USA). Metabolites were sorted in ascending order based on their exact mass, and isomers were indicated in ascending order of retention time.

Although reference standards were not available for confirmation, metabolite abundance was expressed as peak area ratio (PAR, %) with respect to the total peak area (TPA) for each sample. In pHLM, TPA did not include 3-HO-PCE peak area corresponding to the remaining fraction after 2 h incubation, which did not undergo metabolization. However, in biological samples (urine, hair), 3-HO-PCE peak area was included in the calculation of total peak area as it represented the fraction of unchanged 3-HO-PCE excreted in urine or as a parent drug integrated into the hair shaft. 

To cross-check the metabolites identified, raw data from biological samples (urine, hair) analyzed by LC-HRMS were processed using the same metabolic workflow as pHLM incubates. Drug-free urine and hair samples were analyzed and processed simultaneously to subtract background signals and avoid false positive results. 

Since commercial reference standards of the detected metabolites were not available, NMR spectra could not be acquired, thus structure assignments are tentative, based on ddMS2 fragmentation. ddMS2 spectra and extracted ion chromatograms of 3-HO-PCE and its related metabolites obtained from HLM, urine or hair are reported as Supplementary Material (Figures S2 and S3, respectively). 

## 3. Results

### 3.1. Identification of 3-HO-PCE by NMR

1H and 13C and 2D NMR data confirm the structure. The chemical shifts of aromatic protons are characteristic of a meta-substituted phenol. The signal at 4.85 ppm is water brought by the deuterated methanol (MeOD) and by the product itself. The signal at 3.32 ppm is that of MeOH. 3-HO-PCE NMR spectra are presented in Supplementary Material Figure S1.

### 3.2. Metabolism of 3-HO-PCE

Overall, 15 putative metabolites of 3-HO-PCE were identified. Five metabolites had 2 to 4 additional isomers (same exact mass and different retention times), bringing the total detected features to 23. 

**Figure 2:**
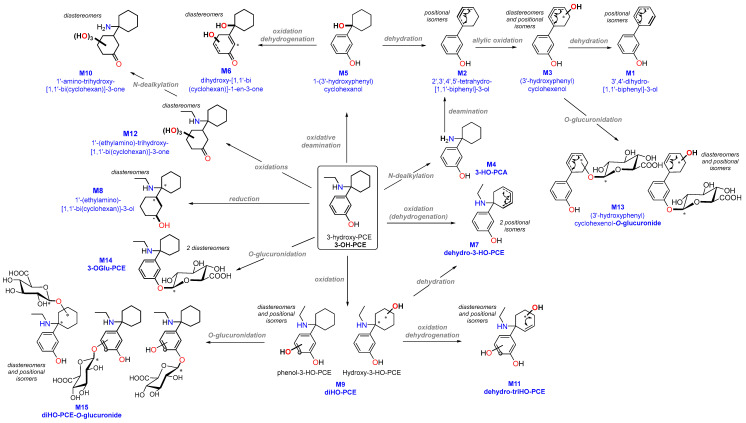
Metabolic pathways of 3-HO-PCE in vitro (HLM) and in vivo (urine, hair). *: creation of stereocenter that explains the identification of stereoisomers.

Figure 2 depicts the suggested metabolic pathway scheme that combines all metabolites of 3-HO-PCE found in pHLM, urine or hair.

Table 1 provides a summary of the names, formulas, exact masses with relative errors, retention times, peak area ratios (PAR) in HLM, urine and hair, and common product ions that can be used for the screening of 3-HO-PCE and its metabolites.

#### 3.2.1. Metabolites Detected in Human Liver Microsomes (HLM)

Out of the 23 detected features, 15 (65%) were identified in HLM, corresponding to 9 metabolites and 6 isomers (M2a, M2b, M3, M7, M9, M11, M12, M13a, M13b, M13c, M14, M15a, M15b, M15c, M15d). Five of these metabolites were found in patient samples, with M2b and M11 present in urine and M2a, M7, and M9b present in hair; none were detected simultaneously in both biological matrices.

The most commonly detected metabolites in HLM were M2a (70.2%), M14 (3-OGlu-PCE) (21.6%), and M3 (5%), while the remaining metabolites were less abundant (≤1%). Only M2a was found in patient hair at a low prevalence (<1%).

Seven metabolites, including one isomer, were present in patient urine (M6b, M9a), hair (M1, M4, M5, M6a, M8), or both (M10), but were not detected in HLM. 

#### 3.2.2. Metabolites Detected in Biological Samples

- Urine

Five metabolites were detected in urine, with two of them confirmed in HLM (M2b, M11), and one found in patient hair (M10). The remaining two metabolites (M6b, M9a) were exclusively present in urine. 

M2b was the most abundant metabolite detected in urine, accounting for 78.8% of the metabolites found. In contrast, M2a, a positional isomer was the most prevalent metabolite detected in HLM. Following M2b, the next most prevalent metabolites in urine were M10 (11.2%), M9a (4.8%), and M6b (3.7%). A diastereomer of M6b, known as M6a, was found in hair at a similar level (3.4%).

- Hair

A total of 9 metabolites were detected in patient hair, with 3 of them confirmed in HLM (M2a, M7, M9b), and one found in urine (M10). The remaining 5 metabolites (M1, M4, M5, M6a, M8) were exclusively present in hair.

M7 was the most prevalent metabolite found in hair, accounting for 48% of the total, followed by M5 (26.3%) and M4 (8.9%). Of the three metabolites detected in hair, only M7 was present in HLM in low amounts (<1%).

**Figure 3:**
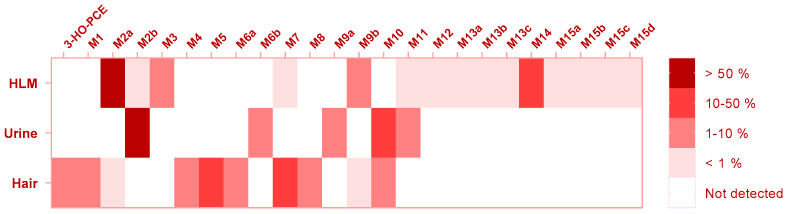
Heat Map-Based visualization of 3-HO-PCE metabolic profile and Abundance Estimation in HLM Urine, and Hair.

The heat map in Figure 3 depicts the detected metabolites arranged by their abundance (Peak Area Ratio PAR, %) across the various analyzed samples. 

## 4. Discussion

The metabolism of 3-HO-PCE closely resembles that of O-PCE (*N*-ethyl-deschloroketamine), which is another designer ketamine analog belonging to the eticyclidine family that we have previously studied [16]. 

It is important to note that the metabolites detected in biological samples but absent in human liver microsomes (HLM) may be attributed to the absence of certain metabolic enzymes, which are not present in HLM as it only represents the microsomal components of hepatocytes. Alternatively, extrahepatic metabolism of 3-HO-PCE may also play a role in the observed metabolite profile.

The phase I metabolism of 3-HO-PCE involves a variety of reactions, mostly *N*-dealkylation, deamination and oxidation, leading to the creation of a total of 12 metabolites. The remaining 3 metabolites are phase II O-glucuronides on either the phenyl ring or the 6-membered carbocycle. 

One of the primary metabolic pathways for arylcyclohexylamine derivatives is *N*-dealkylation, which results in the formation of norketamine, normethoxetamine, or *O*-PCA from ketamine, methoxetamine, or O-PCE, respectively [16,19,29]. In the case of 3-HO-PCE, this pathway has been implicated in the production of 3-HO-PCA (M4) from 3-HO-PCE, as well as M10 from M12. However, M4 and M10 were not detected in HLM, likely because the former is an intermediate metabolite and a precursor to the most prevalent metabolite (M2), while the latter is an end-product of a less prevalent precursor (M12). It is important to note that M4 and M10 were detected in significant amounts in patient urine and hair, respectively. 

During the metabolization of 3-HO-PCE, both oxidative and non-oxidative deamination reactions were observed, resulting in the formation of M2 and M5, which are among the most frequently detected metabolites in urine and hair, respectively. It is noteworthy that while oxidative deamination was not previously reported for ketamine and MXE, it has been described as one of the most prevalent reactions of O-PCE metabolization, accounting for up to 72% of the total peak area in HLM [16,29].

Ketamine and its designer derivatives are frequently hydroxylated [16,19,29]. Although this reaction was not a major metabolic pathway, 5 phase I hydroxylated metabolites of 3-HO-PCE (M3, M6, M9, M11 and M12) have been identified in the present study. The comparison between 3-HO-PCE and O-PCE metabolic profiles allowed to identify common hydroxy- metabolites or isomers based on the position of the OH- group on the phenyl ring or the cyclohexane group (Table 2):

Upon comparison, a discernible metabolic association is apparent between the two eticyclidine derivatives. Specifically, M9a (2-OH-PCE), which is a metabolite of O-PCE, is an isomer of 3-HO-PCE, leading to potential confusion between the two compounds under the present analytical conditions due to their simultaneous elution. The remaining metabolites also exhibit this trend.

The most prevalent eticyclidine derivative metabolite found in HLM was M2a (70.2%), which has a positional isomer, M2b, relating to the double-bond location on the cyclohexane group. M2b was the most commonly detected metabolite in patient urine, accounting for 78.8% of the total. Similarly, the isomer of M2a, M3, was the second most prevalent metabolite in urine derived from O-PCE metabolism, representing 16% of the total [16]. The results obtained indicate that M2b and other frequently identified isomers can be useful biomarkers for untargeted screening of the eticyclidine group in urine samples.

In hair, our previous research has suggested that 2-OH-PCA is a biomarker for O-PCE exposure [16]. In the case of 3-HO-PCE intake, it was found that 3-HO-PCA accounted for a substantial amount (8.9%) of the patient’s hair sample, indicating its potential usefulness as a biomarker for 3-HO-PCE exposure. These results further support the use of 3-HO-PCA as a reliable biomarker for untargeted screening of the eticyclidine family in hair (Table 3). 

For targeted screening of eticyclidine derivatives, two potential strategies may be pursued: firstly, the use of a more efficient column for chromatographic separation of isomers, and secondly, the exploration of specific metabolites for each compound. These approaches are particularly crucial in situations where the parent drug is undetectable in biological samples, as demonstrated in this study where 3-HO-PCE was not detected in patient urine. In such cases, M10 (*m*/*z* 241.13141) is recommended as the target analyte for 3-HO-PCE screening in urine. For the long-term monitoring of 3-HO-PCE exposure, M5 (*m*/*z* 192.11503) presents a promising candidate for hair analysis (Table 3). 

For phase II metabolization, the second most significant 3-HO-PCE metabolite detected in HLM was 3-OGlu-PCE (21.6%). Unfortunately, it was not detected in the urine of the patient, possibly due to the time gap between the last use and urine collection (unknown here) or the short elimination half-life. Additionally, the localization of UGT enzymes in the internal membrane of the endoplasmic reticulum could have hindered the in vivo access of substrates, contributing to the phenomenon known as “latency,” which makes it challenging to extrapolate in vivo effects of UGT enzymes from in vitro experiments using isolated microsome tissues [30]. Moreover, as glucuronides are hydrophilic compounds, their integration into the hair shaft is less important than the parent drug. Hence, it may not be possible to detect some of these metabolites in hair [28]. Finally, like ketamine and methoxetamine, *N*-glucuronidation of 3-HO-PCE was not observed, in contrast to what has been seen with O-PCE. Only *O*-glucuronides of 3-HO-PCE or its nor- and hydroxylated metabolites have been detected in the current study [16,18,19,29].

## 5. Conclusions

This study sheds light on the limited knowledge surrounding 3-HO-PCE, a new psychoactive substance belonging to the arylcyclohexylamine class. By exploring its metabolism using Human Liver Microsomes (HLM) and analyzing urine and hair samples from a drug user, this study identified 15 putative metabolites, with 12 produced through phase I metabolism and 3 through phase II *O*-glucuronidation. Notably, this study found that the metabolism of 3-HO-PCE is similar to that of O-PCE, another designer ketamine analog of the same eticyclidine family. The identification of M2a and 3-HO-PCA as reliable biomarkers for untargeted screening of the eticyclidine family in urine and hair, respectively, and the promising candidates M10 and M5 for targeted screening of 3-HO-PCE in urine and hair, respectively could provide valuable information for the detection and identification of 3-HO-PCE in cases of overdose or drug abuse. 

These findings may also aid in the development of new strategies for drug screening and detection, as well as provide important information for clinicians, forensic scientists, and public health officials in their efforts to manage the risks associated with 3-HO-PCE and other designer ketamine derivatives.

## 6. Limitations of the Study

The study has several limitations that need to be taken into consideration. Firstly, the results of the urine analysis should not be used to draw conclusions about the general excretion profile of 3-HO-PCE in humans due to the unknown amount and time of consumption of the drug. Secondly, the lack of reference standards made it challenging to validate and quantify the detected metabolites. 

It is important to note that the patient’s potential consumption of both 3-HO-PCE and O-PCE cannot be ruled out, as some of the identified metabolites may have originated from either substance. Therefore, the current findings should be considered preliminary, and further investigation is necessary to confirm the results. Given that only one authentic sample was analyzed, additional research is required to establish the validity of these findings.

In summary, while this study provides important insights into the metabolic pathways of 3-HO-PCE, the limitations mentioned above should be taken into account when interpreting the results.

## Figures and Tables

**Table 1:** Names, formula, theoretical and observed exact mass, mass error, retention times, peak area ratio in HLM, urine and hair and common product ions of 3-HO-PCE and its potential metabolites.

ID	Name	Formula	Theoretical *m*/*z*	Observed *m*/*z*	Mass Error (ppm)	Isomers	Rt (min)	HLM_PAR_ (%)	Urine_PAR_ (%)	Hair_PAR_ (%)	Common Product Ions
3-HO-PCE	3-HO-PCE	C_14_ H_21_ NO	219.16286	219.16231	−2.52	NA	7.11	NA	ND	1.6	81.06994; 107.04918;
M1	3′,4′-dihydro-[1,1′-biphenyl]-3-ol	C_12_ H_12_ O	172.08936	172.08881	−3.21	NA	6.51	ND	ND	4.4	81.06995; 107.0492; 131.0494;
M2	2′,3′,4′,5′-tetrahydro-[1,1′-biphenyl]-3-ol	C_12_ H_14_ O	174.10501	174.10447	−3.10	M2a	7.11	70.2	ND	<1	79.05428; 81.06996; 107.0492; 133.06494; 175.11192
						M2b	7.84	<1	78.8	ND	
M3	(3′-hydroxyphenyl) cyclohexenol	C_12_ H_14_ O2	190.09992	190.09938	−2.84	NA	6.049	5	ND	ND	79.05424; 81.06996; 95.04906; 131.04919; 145.06494
M4	1-(3′-hydroxyphenyl) cyclohexanamine 3-HO-PCA	C_12_ H_17_ NO	191.13156	191.13101	−2.87	NA	11.68	ND	ND	8.9	67.0544; 94.0652; 119.04933
M5	1-(3′-hydroxyphenyl) cyclohexanol	C_12_ H_16_ O_2_	192.11557	192.11503	−2.81	NA	13.15	ND	ND	26.3	67.0545; 79.05430; 81.06991; 121.0650
M6	dihydroxy-[1,1′-bi(cyclohexan)]-1-en-3-one	C_12_ H_18_ O_3_	210.12614	210.12559	−2.61	M6a	7.85	ND	ND	3.4	67.0544; 81.07001; 121.06490
						M6b	13.4	ND	3.7	ND	
M7	dehydro-3-HO-PCE	C_14_ H_19_ NO	217.14721	217.14666	−2.53	NA	6.97	<1	ND	48	67.0544; 131.04941; 145.06480; 173.09644
M8	1′-(ethylamino)-[1,1′-bi(cyclohexan)]-3-ol	C_14_ H_27_ NO	225.20981	225.20926	−2.44	NA	15.72	ND	ND	4.2	68.04974; 70.0653; 110.09655
M9	phenol- or hydroxy-3-HO-PCE = diHO-PCE	C_14_ H_21_ N O_2_	235.15777	235.15723	−2.29	M9a	2.59	ND	4.8	ND	67.05441; 81.0698; 95.0492
						M9b	5.95	1.2	ND	< 1	
M10	1′-amino-trihydroxy-[1,1-bi(cyclohexan)]-3-one	C_12_H_19_NO_4_	241.13195	241.13141	−2.23	NA	9.25	ND	11.2	2.62	79.05410; 81.06995; 136.07549
M11	dehydro-triOH-PCE	C_14_ H_19_ NO_3_	249.13704	249.13649	−2.20	NA	7.48	<1	1.5	ND	100.11208; 132.0608; 147.0799; 161.0835; 174.12802; 202.12263
M12	1′-(ethylamino)-trihydroxy-[1,1′-bi(cyclohexan)]-3-one	C_14_ H_25_ NO_4_	271.17890	271.17836	−1.99	NA	6.17	<1	ND	ND	67.0543; 81.06995;
M13	1′-(ethylamino)-trihydroxy-[1,1′-bi(cyclohexane)]-dione (3′-hydroxyphenyl)cyclohexenol-*O*-glucuronide	C_18_ H_22_ O_8_	366.13201	366.13147	−1.47	M13a	5.18	<1	ND	ND	79.05417; 81.06996; 131.04993; 173.09573
						M13b	5.85	<1	ND	ND	
						M13c	8.20	<1	ND	ND	
M14	3-HO-PCE-O-glucuronide = 3-OGlu-PCE	C_20_ H_29_ NO_7_	395.19495	395.19440	−1.39	NA	5.97	21.6	ND	ND	81.0699; 107.04915; 133.0646 175.11226;
M15	diOH-PCE-*O*-glucuronide	C_20_ H_29_ NO_8_	411.18986	411.18932	−1.31	M22a	0.58	<1	ND	ND	81.0698; 123.04411
						M22b	3.97	<1	ND	ND	
						M22c	5.83	<1	ND	ND	
						M22d	6.87	<1	ND	ND	

_PAR_: peak area ratio; ND: not detected; HLM: Human Liver Microsomes; Rt: Retention Time.

**Table 2:** Overlapping hydroxy metabolites or isomers in 3-HO-PCE and O-PCE metabolism.

*m/z*	3-HO-PCE Metabolism (Present Study)	Rt (min)	O-PCE Metabolism (Previous Study)	Rt (min)
172.08881	M1	6.51	M2b	6.51
174.10447	M2a	7.11	M3b	7.06
191.09938	3-OH-PCA (M4)	11.68	2-OH-PCA (M5a)	11.67
217.14666	Dehydro-3-OH-PCE (M7)	6.5	O-PCE (Parent drug)	6.5
219.16231	3-HO-PCE (Parent drug)	7.11	M9a (2-OH-PCE)	7.06
235.15723	DIOH-PCE (M9b)	5.95	DIOH-PCE (M12a)	5.82

**Table 3:** Proposed candidate metabolites for untargeted screening of eticyclidine derivatives and targeted screening for 3-HO-PCE in urine and hair.

Biological Sample	Untargeted Screening Metabolites of the Eticyclidine Derivatives	Targeted Metabolite of 3-HO-PCE
Urine	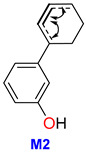 *m*/*z* 174.10447	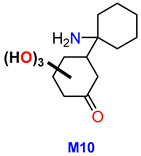 *m*/*z* 241.13141
Hair	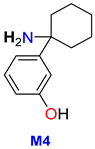 *m*/*z* 191.09938	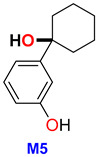 *m*/*z* 192.11503

## Data Availability

The data presented in this study are available on request from the corresponding author. The data are not publicly available due to medical confidentiality.

## Supplementary Materials

The following supporting information can be downloaded at: https://www.mdpi.com/article/10.3390/metabo13030432/s1, Figure S1: ^1^H NMR (top) and ^13^C NMR (bottom) spectra of 3-HO-PCE; Figure S2: Extracted chromatograms of 3-HO-PCE and its potential metabolites from HLM, urine or hair; Figure S3: Full scan (Top) and ddM2 (Bottom) High resolution mass spectra of 3-HO-PCE and its putative metabolites.
