# Supplementary material for: Characterization of 3-Hydroxyeticyclidine (3-HO-PCE) Metabolism in Human Liver Microsomes and Biological Samples Using High-Resolution Mass Spectrometry
Source: Metabolites. 2023 Mar 16;13(3):432. doi: 10.3390/metabo13030432 (PMC10055977; doi:10.3390/metabo13030432)
Supplement: Supplementary file 1 [file metabolites-13-00432-s001.zip › metabolites-2262443-supplementary.pdf]

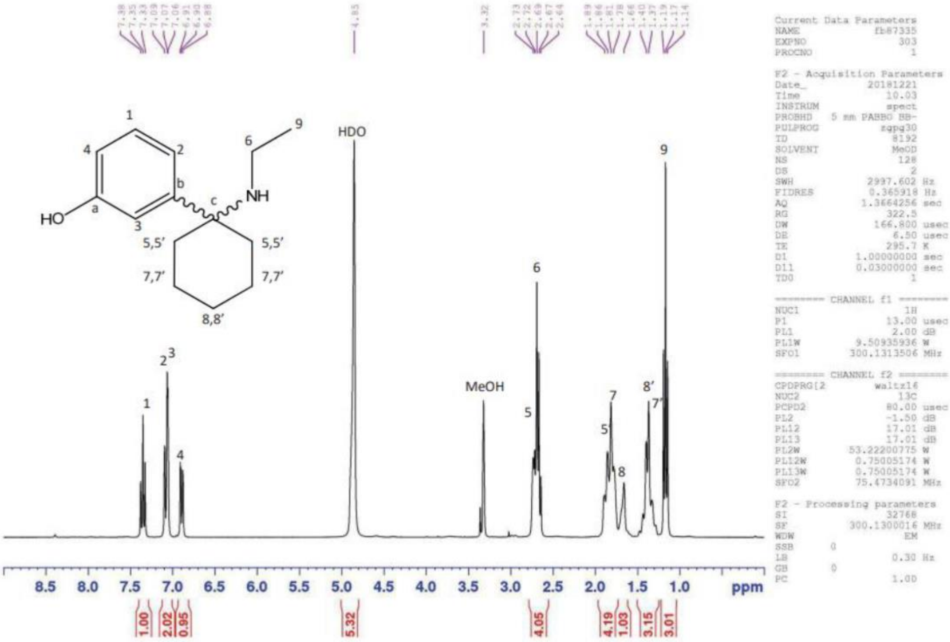

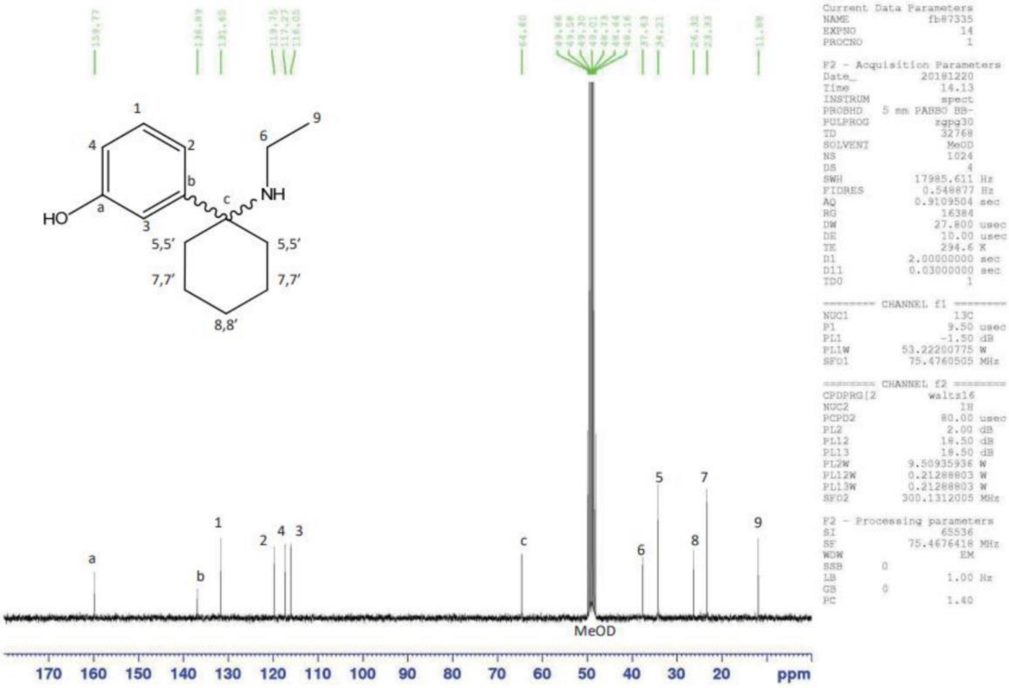

Figure S1. ^{1}H NMR (top) and ^{13}C NMR (bottom) spectra of 3-HO-PCE.

3-HO-PCE

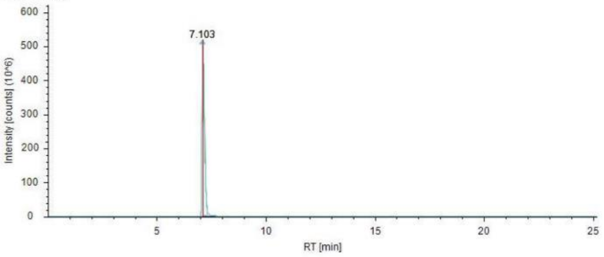

M1

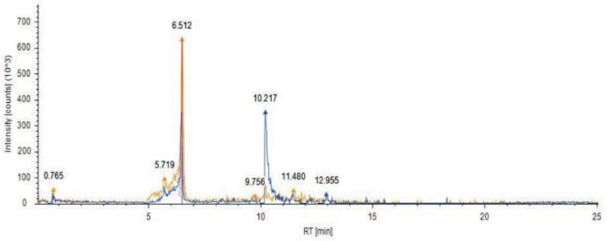

M2 :

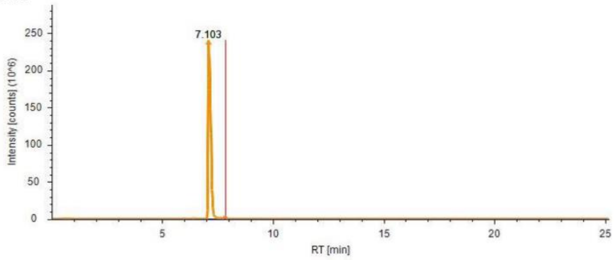

M3

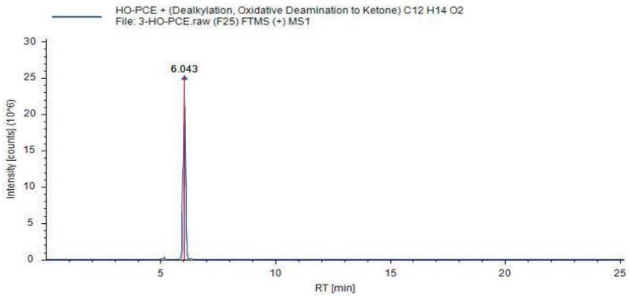

M4

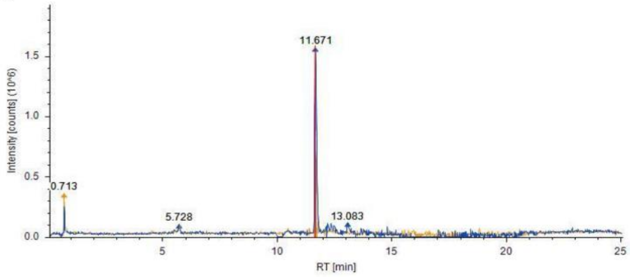

M5

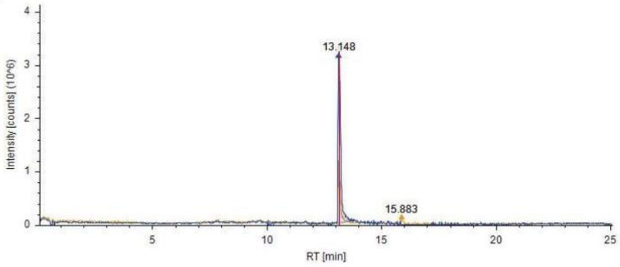

M6a

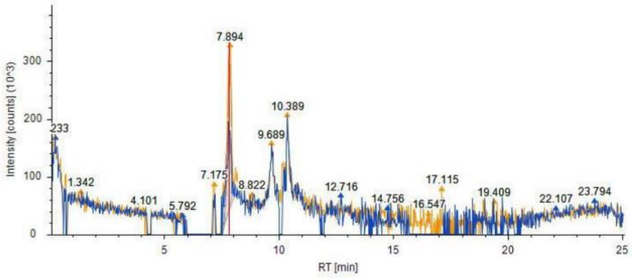

M6b

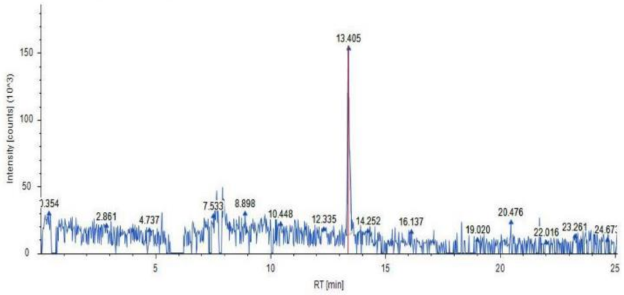

M7

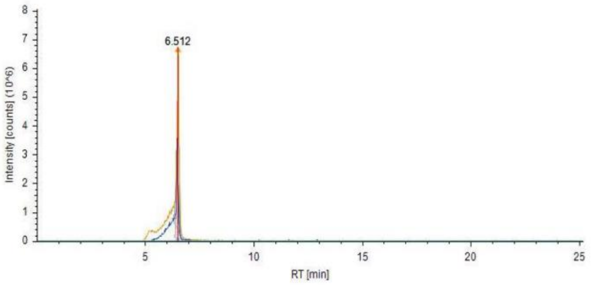

M8

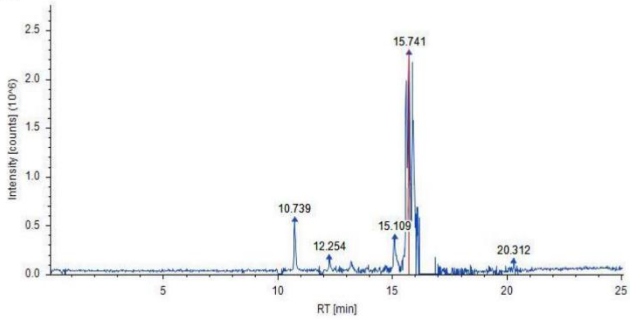

M9a

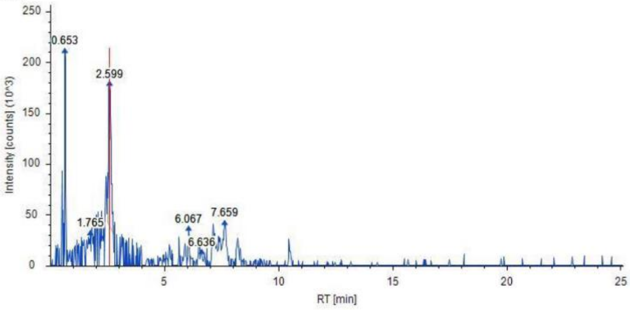

M9b

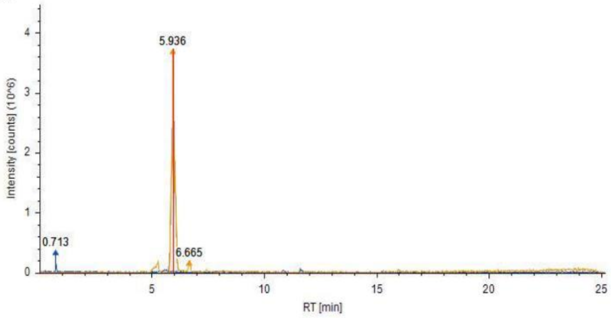

M10

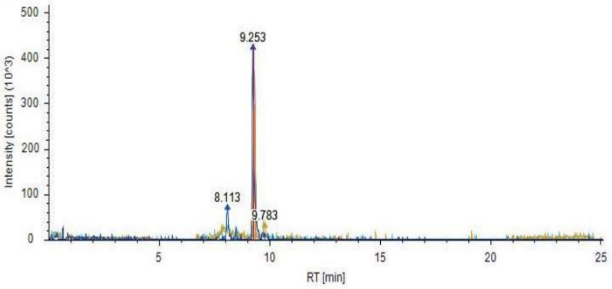

M11

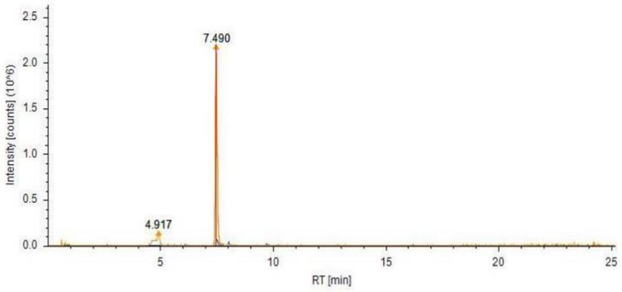

M12

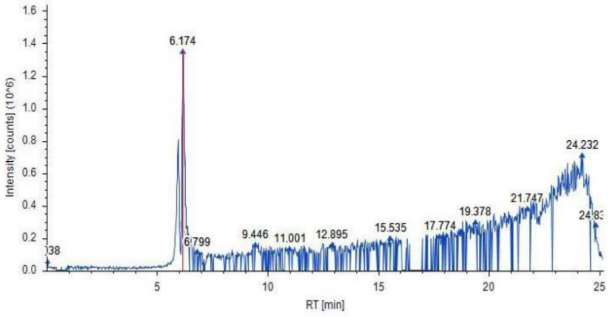

M13

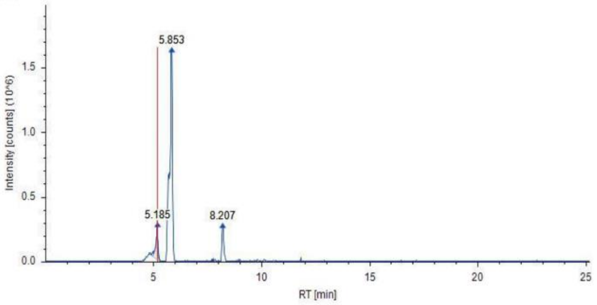

M14

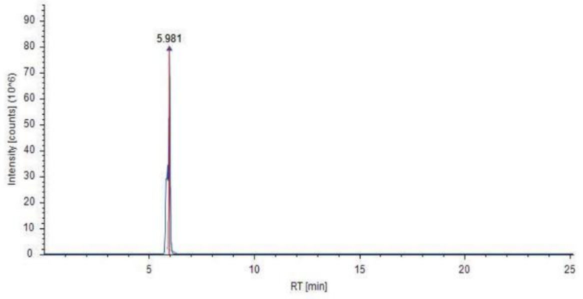

M15

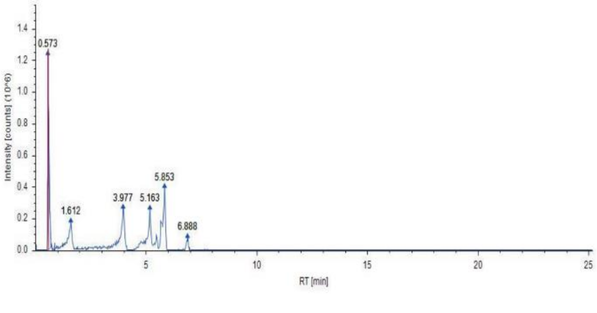

Figure S2. Extracted chromatograms of 3-HO-PCE and its potential metabolites from HLM, urine or hair.

Putative structure elucidation proposed by Compound Discoverer software on each ddMS2 spectra was consulted but not used to build the metabolic profile of 3-HO-PCE, and is provided here for informational purposes only.M6b did not trigger a ddMS2 fragmentation due its low abundance.

3-HO-CPE

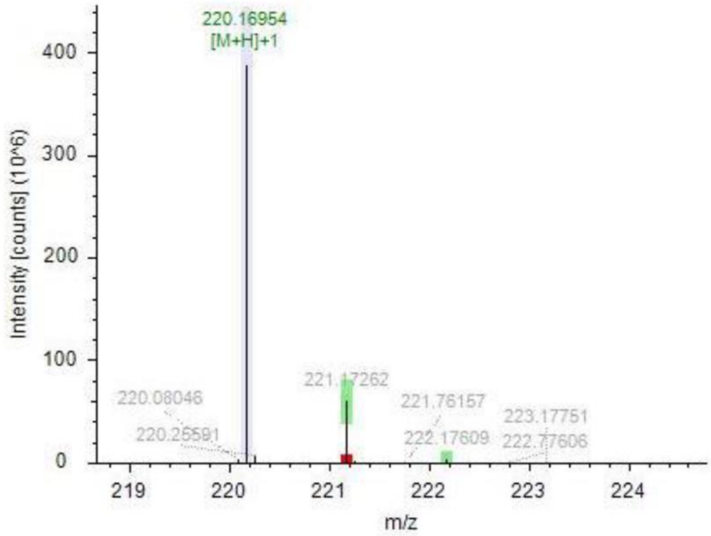

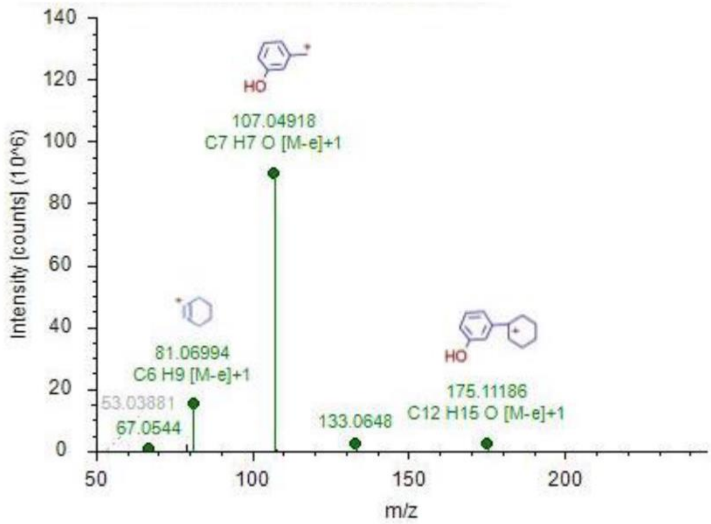

Figure S3. Full scan (Top) and ddM2 (Bottom) High resolution mass spectra of 3-HO-PCE and its putative metabolites.
